# Siamese Neural Network for Keystroke Dynamics-Based Authentication on Partial Passwords

**DOI:** 10.3390/s23156685

**Published:** 2023-07-26

**Authors:** Kamila Lis, Ewa Niewiadomska-Szynkiewicz, Katarzyna Dziewulska

**Affiliations:** 1 Research and Academic Computer Network, Kolska 12, 01-045 Warsaw, Poland; kamila.lis@nask.pl (K.L.); katarzyna.dziewulska@nask.pl (K.D.); 2 Institute of Control and Computation Engineering, Warsaw University of Technology, Nowowiejska 15/19, 00-665 Warsaw, Poland

**Keywords:** keystroke dynamics, behavioral biometry, keyboard, partial password authentication, siamese network

## Abstract

The paper addresses issues concerning secure authentication in computer systems. We focus on multi-factor authentication methods using two or more independent mechanisms to identify a user. User-specific behavioral biometrics is widely used to increase login security. The usage of behavioral biometrics can support verification without bothering the user with a requirement of an additional interaction. Our research aimed to check whether using information about how partial passwords are typed is possible to strengthen user authentication security. The partial password is a query of a subset of characters from a full password. The use of partial passwords makes it difficult for attackers who can observe password entry to acquire sensitive information. In this paper, we use a Siamese neural network and n-shot classification using past recent logins to verify user identity based on keystroke dynamics obtained from the static text. The experimental results on real data demonstrate that keystroke dynamics authentication can be successfully used for partial password typing patterns. Our method can support the basic authentication process and increase users’ confidence.

## 1. Introduction

User authentication enforces secure access to computer systems, networks, and devices. The motivation to authenticate users ranges from access control reasons to business development purposes such as adding e-commerce, e-banking, etc. In recent years, the challenges related to identifying users and ensuring secure access to their accounts have increased due to the increasing number of cyber threats. Therefore, it is recommended to extend passwords to make them more challenging to crack [1]. Meanwhile, most people need to take precautions to secure their accounts. Persuading users to set strong passwords is needed. They often use simple passwords and do not use two-factor authentication for their accounts. Many people use the same password for most of their accounts. In contrast, attackers launch more and more phishing URLs every year. Phishing targets a user’s authentication rights and identity.

Nowadays, it is clear that passwords are no longer the only approach to user authentication. There is a growing demand for different types of technologies for user identification, both online and in physical systems. As technology advances, authentication mechanisms are constantly being improved. Many authentication technologies and an even greater range of activities require authentication methods.

A standard security mechanism is the addition of authentication steps, most often requiring the rewriting of a secret transmitted through another communication channel. With frequent logins, such an extended process can persist for the user. However, it is possible to conduct additional verification without introducing additional interaction with the user, but only by observing their behavior. It has been proven that the keystroke pattern of an individual is unique, in a similar manner as a handwritten signature [2]. In this way, when logging in, the typing dynamics can also be analyzed, in addition to verifying the correctness of the password. In addition, in the event of password compromise, device takeover, or remote control, behavioral biometrics allows abnormalities in user behavior to be observed. It is also worth mentioning that implementing algorithms based on behavioral biometrics is simple, as they do not require any additional hardware components.

Although the potential uses of keystroke dynamics remain strong, it still needs to be widely deployed in many security applications. Unlike the stability offered by iris or retinal recognition, there is no permanence in the specific typing pattern of an individual. Typing rhythm may change daily due to several factors, such as physical fatigue, lack of attention when typing, or even using a different computer keyboard. Data acquisition is challenging in an uncontrolled environment. Users can use different machines, virtual keyboards, smartphones, etc. For each device, the collected data are slightly different. Furthermore, poorer quality equipment can degrade the accuracy of the result. Maxion et al. [3] claim that artifacts injected by a USB keyboard can change an algorithm’s decision by nearly 20 percentage points. In addition, there may also be some legal issues or user concerns about the security of their data. These concerns are analogous to other biometrics that are nevertheless widely accepted (e.g., unlocking a phone with a fingerprint).

As noted in [4], most methods based on behavioral biometrics usually have three main drawbacks: (i) they need lengthy interactions (minutes), or a very long time to collect the passphrase samples, to learn the user behavior, (ii) they require ad-hoc interaction challenges or (iii) need a model per user to improve model accuracy.

Despite the disadvantages and challenges mentioned above, biometric methods are increasingly used in authentication systems. It is due to their numerous advantages. These include:

- Uniqueness—everyone writes differently;
- Low implementation and deployment costs—no need to provide the user with additional equipment and easy integration of additional modules into commonly-used authentication systems;
- Transparency;
- Provision of additional security mechanism;
- Possibility of continuous monitoring.

It is also worth mentioning that The New York State DMV and the European Banking Authority approve typing biometrics as a compliant identity authentication method [5].

In this paper, we propose the usage of keystroke dynamics on static passwords to support the basic authentication process. While much research focused on keystroke dynamics authentication on a full password, we claim that this mechanism can also be used for partial password login procedures. The main benefit of the partial password mechanism is that it is an easy way to impede attackers who can observe password entry by shoulder surfing, keylogging, or browser malware. It prevents from revealing of complete information in a single step. Partial passwords are a standard security feature in the financial sector [6]. Hence, the usage of the partial password increases logging security. However, it challenges keystroke dynamics-based authentication.

To sum up, this work’s main contribution is to present the multi-shot keystroke dynamics-based classification algorithm employing a Siamese neural network (SNN) model trained on historical data gathered from a group of users and a set of recent logins of a user for verifying his identity. We have compared the accuracy of user verification based on full (complete) and partial (masked) password typing patterns, and we tested the sensitivity of the proposed method to SNN architecture and model parameters. Moreover, we present a self-developed software environment for real data about password typing dynamics acquisition.

In our solution, we deliberately did not use a keycode. The information about entered characters allows us to compare time distances for *n*-grams (or *n*-graphs). Such an approach can be used for free and fixed text with typos. However, collecting credentials for security reasons is questionable. The collection of such data would understandably make users distrustful of our solution. Given the advantages and disadvantages of using a keycode as a feature, we decided not to use it for the classification process.

The rest of the paper is structured as follows. Section 2 presents the survey of the application of user keystroke dynamics to user authentication. Section 3 provides a formulation of the problem to be solved. Our artificial neural network architecture for user authentication is overviewed in Section 4. The software for real data acquisition, training, and validation setups and results are described in Section 5. The results of the performance evaluation of the SNN-based classification algorithms for full and partial-password-based authentication are presented and discussed in Section 6 and Section 7. Finally, Section 8 concludes the paper and highlights future research directions.

## 2. Related Works

Analysis of keystroke dynamics, similarly to other biometrics such as voice or written text analysis, can be broadly classified into two types—*static* (structured text) and *dynamic* (free text) [7]. Static analysis involves analyzing the keystroke behavior of an individual on a predetermined phrase at certain points in the system. In authentication systems based on login and password, such predefined phrases are the user’s login data. It can also involve the use of a particular phrase that is common to all users. The user’s typing pattern is analyzed only at this stage of interaction with the system. Static text analysis can be deployed, especially in systems without further text entries. Dynamic analysis involves continuous or periodic monitoring of keystroke behavior. The analysis starts at login and continues the entire time one uses the system. Compared with fixed-text keystroke dynamics, the free-text case presents some additional challenges. First, the number of valuable features may differ among input sequences. Second, the optimal length of a keystroke sequence for analysis is a factor that must be considered—a more extended sequence is slower to process and might include more noise. In comparison, a shorter sequence may need more features to be considered.

The idea of using information about typing rhythm on a keyboard to authenticate a user has been developing for many years. Its basis lay in the 1897 observation when it was noticed that telegraph operators have distinctive patterns of keying messages over telegraph lines and can recognize their fellow workers based on their typing rhythms [8]. This method of identifying the sender of the telegraph by using the rhythm, pace, and syncopation of the telegraph keys, known as the “Fist of the Sender”, was also valuable during World War II [9].

Over the past 40 years, it has been used in many methods for static analysis of user keystroke dynamics. The first attempts were based on a simple statistical approach. The mean and the standard deviation were computed for comparison using hypothesis testing by authors of [10,11]. In other research papers, various distance measures were used [12,13,14]. In the 1990s, widely used solutions employed statistical estimation of the distance between vectors of delays between keyboard events. The topic of keystroke dynamics authentication gained significant popularity in the 2000s [15]. In subsequent years, techniques using clustering methods, different distance estimations, measures of the randomness of intervals (for free text), decision trees, and artificial neural networks have been developed and tested. Since 2010 measures of disorder, Hidden Markov models, probability density estimates, and machine learning (SVM, Random Forest) have also been used.

Siamese neural network, a unique artificial neural network, has received much attention recently. In [16], authors investigated the feasibility of using a Siamese network for keystroke authentication on full passwords. They developed and trained the Siamese network model on 200 samples per user taken from the CMU dataset [14], collecting data on the typing dynamics of full passwords by users who entered the same password on the same machine. They experimented with various network architectures, pre-trained models, and attention obtaining satisfactory results. The authors claim they obtained 90.8% user authentication accuracy after 30 logins. Another tool using the Siamese network for user authentication based on his few previous logins is described and evaluated in [4]. Logins on both workstations and smartphones were considered. The authors considered different human-computer and human-smartphone interaction features, i.e., keystroke and mouse dynamics, holding patterns, and touch patterns. The method was tested on a database that contained over 100 K different web interactions collected in the wild. Unfortunately, this dataset is not publicly available online. The user authentication accuracy achieved after 30 logins was equal to 88%.

Generally, the method considered should be adjusted to the size and quality of the available experimental dataset. The comparative study of two commonly used statistical algorithms: the scaled Manhattan and the Instance-based Tail Density (ITAD) metrics [17], with the state-of-the-art deep learning model TypeNet [18] on small and large datasets, is presented and discussed in the paper [19]. The results serve as a reminder of the general intuition—deep neural networks produce better results when trained on a sufficiently large dataset, whereas when the learning dataset is undersized simple statistical algorithms perform better.

The usability of keystroke dynamics-based authentication was analyzed in [20], where it serves as a second factor in multi-factor authentication. The scaled Manhattan distance was computed for signup, login (username and full password), and account recovery, which required an additional predefined text. The authors propose employing an OTP factor (one-time password) until a sufficient number of samples have been collected and after the enrollment process is completed whenever keystroke-based authentication fails.

In conclusion, while much research focused on keystroke dynamics-based authentication on full passwords, there is a lack of similar works with partial passwords. One of the reasons may be the lack of publicly available datasets with partial passwords. Collecting such a dataset is a very cumbersome process. Moreover, developing an authentication method based on keyboard dynamics for partial passwords is a challenge.

## 3. Problem Formulation

**Figure 1:**
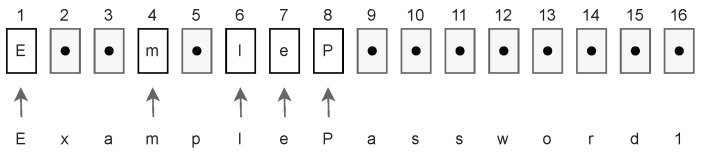
Filling partial password.

Suppose a user logs into his account by entering a partial password. A partial (masked) password is a query of a subset of characters from a full password, as presented in Figure 1.

The aim is to develop a method for static analysis of user keystroke dynamics that can be used to support his authentication. The problem boils down to online checking the similarity between the dynamics of the partial password being entered and the patterns gathered in the user’s previous logins and detecting potential fraud.

Applying behavioral biometrics to the partial password is a challenging task. When a user types a full password, the phrase he enters is always the same. With no outside disturbance, he enters the password automatically. Differences in periods are relatively small, making creating a consistent user behavioral model easy. In a partial password, the following phrase to enter can be a different combination of characters from the password, so entering it can not be done automatically. The user often has to think about which character to enter next. Because the combinations repeat themselves randomly, it is highly probable that, for an identical combination, their hesitation will affect different signs. For this reason, it is hard to create a user behavioral model for partial passwords.

Another problem arises from many combinations of the partial password on the basis on which the user has to be recognized. Searching for the same combination as the user entered to authenticate him is time-consuming and computationally expensive. Therefore, fast and efficient models and tools for partial-password-based authentication are welcome.

Let us assume that a sequence of the following data describes a single login:

- User ID;
- Username;
- Login counter;
- Information regarding whether a password is partial or completed;
- Character positions in a password;
- Keypress timestamps.

**Figure 2:**
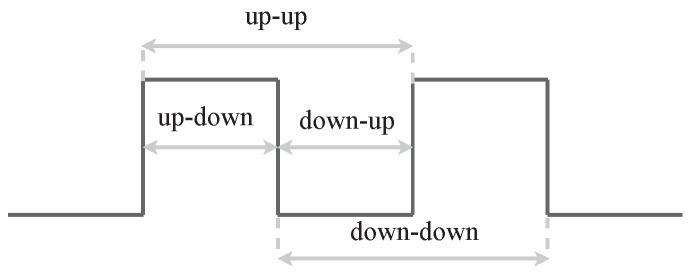
Recorded time between key events.

No information on what key was pressed can be collected for privacy reasons. The only available data are concerned with pressing and releasing a key. The raw sequences of key presses include distances between timestamps of pairs of following events. To sum up, the following time distances are recorded for each character in the password (see Figure 2):

1. down-up (dwell time)—how long the key is pressed;
2. up-down (speed)—flight time from one key to the next key pressed;
3. up-up—time between key-up events;
4. down-down—time between key-down events.

## 4. Siamese Neural Network Models for Keystroke Dynamics-Based Authentication

### 4.1. Siamese Neural Network

We propose to use machine learning techniques for keystroke dynamics-based authentication. Most commonly, deep machine learning is used to classify based on a learned behavioral model for each user. This approach involves the requirement to collect a sufficiently large learning set. We must rely on something other than obtaining data from numerous logins. Hence, we propose to apply Siamese neural networks (SNN), a model that learns semantic similarity and can be trained on a tiny number of samples per class. Siamese focuses on learning embeddings that place the same classes close together. The ability to learn from very little data has made this network very popular in recent years. They are widely used in verification systems [21], i.e., face recognition, signature verification, etc.

A Siamese neural network [22] comprises two or more identical artificial subnetworks with the same configuration, parameters, and weights. Both subnetworks work parallelly in tandem. Each is capable of learning the hidden representation of a given input sample. The distances between the outputs of these subnetworks are inputs to the decision network. Finally, the loss value is calculated for the decision network outputs. The loss function aims to minimize the intra-class distance and maximize the distance between samples from different classes. Hence, the output generated by a Siamese neural network execution can be considered the semantic similarity between the representation of the two input samples.

Siamese neural network architecture has already been used for keystroke dynamics-based authentication on full passwords and achieved good scores, as is presented in [4,16]. Therefore, we employed a Siamese neural network for user authentication on a partial password. We frame keystroke dynamics-based authentication as a binary classification problem. We calculate the similarity score of the assessed and previously collected user samples (time distances) to distinguish genuine and imposter users. Applying SNN and one-shot learning, only a few samples of data per class are sufficient to recognize the password owner. Moreover, it allows for limiting the number of trained models. The same model can be used for all users, and it does not have to be retrained with the appearance of new data (one-shot learning). To update a model after increasing the number of users, model parameters can be reused based on the similarity between the keystroke dynamics data of old and new users. In general, categories are first learned on numerous training examples, and then new categories are learned using transformations of model parameters from those initial categories.

### 4.2. Snn Architecture Overview

We have developed two Siamese neural network models for keystroke dynamics-based authentication on full and partial passwords. The learning goal of a Siamese network is to minimize a distance metric for similar objects and maximize this metric for distinct ones. In our case, the objects that are compared are keystroke patterns of users. The similar objects denote the keystroke dynamics of the same user’s logins, while the distinct objects denote the keystroke dynamics of various users’ logins.

**Figure 3:**
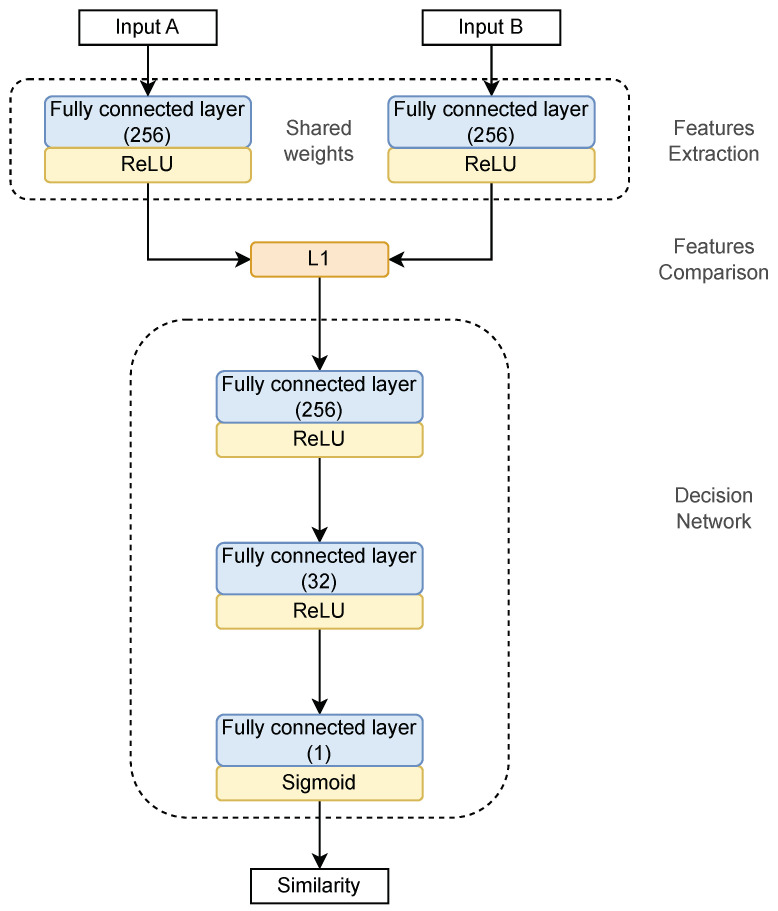
The architecture of the Siamese neural network model.

The network architecture employed in both models is depicted in Figure 3. It consists of three subnetworks. Two identical networks (Siamese network subnets) are responsible for features from the input data extraction. A pair of keystroke dynamics samples, i.e., vectors composed of recorded times presented in Figure 2, is fed to the inputs of these networks. Each subnetwork comprises a single fully connected layer sharing the same architecture and weights. The layer is comprised of 256 neurons with the ReLU activation function. The subnetworks project two login samples onto the same latent space in which these samples can be compared. One of the learning process results is the optimal transformation of input samples into latent space.

Then, the outputs of the two subnetworks, i.e., login samples projected onto the latent space, are compared. The distance vector L1 is calculated:

(1) $$L1\left(y_{s}^{1},y_{s}^{2}\right)=\left|y_{s}^{1}-y_{s}^{2}\right|,$$

where $y_{s}^{1}$ and $y_{s}^{2}$ denote subnetwork outputs (vectors of dimension 256), i.e., projected two login samples.

The third subnetwork (decision network) calculates the similarity score of two login samples based on the L1 distance vector (Equation (1)). It comprises three fully connected layers of 256 and 32 neurons with ReLU activation function and 1 neuron with Sigmoid activation function, respectively. The output of the decision network, i.e., $y_{d}\in \left[0,1\right]$, is the probability of similarities of login samples. It is used for the classification of sample logins. Finally, the input data is classed as *the same user* (class “1”) for $y_{d}\leq \varphi$, and *the other user* (class “0”) for $y_{d}>\varphi$, where φ denotes the assumed threshold.

We implemented our Siamese neural network architecture using the Pytorch [23] framework and Ignite [24] high-level library. We used Binary Cross Entropy as a loss function and Adam optimizer.

Two models for user authentication on full and partial passwords were trained. The models training procedure consisted of the following steps:

1. Initialize the network model, loss function, and Adam optimizer.
2. Pass the first password of the password pair (full or partial) through the network.
3. Pass the second password of the password pair (full or partial) through the network.
4. Calculate the loss using the outputs from the first and second passwords.
5. Pass the final loss through the decision network.
6. Back propagate the loss to calculate the gradients of the model.
7. Update the weights using Adam optimizer.
8. Save the model.
9. Read out the network output.
10. Make the final decision—assign the input data to the appropriate class based on the threshold value.

These steps are repeated until each pair of passwords pass through the network in each training epoch.

## 5. Training and Validation of Classifiers

### 5.1. The Software Tool for Experimental Data Acquisition

A key aspect in the training and validation of artificial neural networks is the preparation of a representative dataset. Many datasets contain data on the typing dynamics of full passwords, such as described in [14,25,26]. According to our knowledge, no datasets contain data on the typing dynamics of partial passwords. Therefore we decided to develop an experimental platform for collecting data concerning partial and full password typing. We have prepared a website simulating a bank login page. Data on the typing dynamics of passwords were collected in an uncontrolled environment, as similar as possible to the production one. Whenever the user pressed or released a key, the web page recorded the event (i.e., key-down or key-up) with its timestamp of occurrence. Distances between those events, depicted in Figure 2, were saved to the database.

The experimental data were collected from 39 volunteers, 10 women and 29 men aged between 23 and 55. Each user was given an assigned username and password. Usernames were unique, while passwords were distributed from the previously prepared fifteen passwords. Eleven passwords were created randomly using Norton Password Manager [27]. Moreover, we set four passwords with ID = 12, 13, 14, and 15 to be more “human-like”. All passwords used in the training process are presented in Table 1.

It should be noted that in the case studies described in the literature, the login credentials are distributed among all users. Hence, all users typed the same passwords. The aim was to provide positive samples that simulated a fraud attempt without requiring users to log into other users’ accounts. It also made it possible to train the network to operate on the time sequences of entering different passwords. However, such an approach is far from reality. In reality, each user has a password unknown to others. Thus, in our case study, each password was assigned to at least three users. Hence, a maximum of three users shared the same password.

The data collection process was divided into 16 sessions, each with 25 logins. Users were asked to replenish 1 session per day. A single login consisted of entering the username and then a password twice, i.e., the full password in a text field and selected characters of that password in separate text fields (partial password). These fields were selected randomly from a given full password. The other fields were blocked. Characters for partial passwords were randomly selected in every logging trial.

In conclusion, similarly to the systems using partial passwords for user authentication, our data acquisition tool required users to enter a different, randomly selected subset of characters each time they logged in. Thus, the password did not change, while the subset of characters the user was asked to enter changed every login.

As the user typed the password, it was checked for correctness. If the user made a typographical error, the application prompted the subject to retype the password. We recorded timestamps for 25 correctly typed full and 25 partial passwords in each session. The correctness of the collected samples was verified. We removed all outliers of time sequences possibly damaged by hardware or software errors. The sequences of keyboard features of the wrong length, containing negative values or values above the established threshold, were discarded. Finally, two datasets containing keystroke dynamics data were created:

- FPdataset: full password data collection consisting of 18,271 samples.
- PPdataset: partial password data collection consisting of 18,259 samples.

### 5.2. Training and Validation Datasets Preparation

The next step was to process data collected in FPdataset and PPdataset and prepare datasets for training and to validate two keystroke dynamics classifiers described in Section 4. Each sequence from FPdataset and PPdataset was scaled to seconds, transformed into a one-dimensional tensor, and fitted to an assumed maximum number of characters (assumed maximal length of a password). In our research, we limited this number to 16 characters.

As SNN learns to define the difference between two input data, the training and validation datasets are collections of pairs of samples. In our research, it comprises pairs of time sequences taken from the FPdataset and PPdataset, respectively. Table 2 presents the example pairs of samples.

Finally, for each classifier, we have created two datasets of pairs, respectively, each containing samples of (i) the same user and (ii) different users, according to the Algorithm 1.
**Algorithm 1** Samples pairs generation1:Group users by the password they type (*k* - number of groups, $n_{k}$ - number of users in the group *k*)2:**for** each group *j* (j=1,…,k) **do**3:   **for** each user in a group *i* ($i=1,\ldots ,n_{k}$) **do**4:     pair the samples from the logins of the same user *i* and assign them the label 15:     pair the samples from the user *i* with randomly selected samples from other users     m≠i in the group *k* and assign them the label 06:   **end for**7:**end for**

To create training and validation datasets, we divided FPdataset and PPdataset into two subsets, according to the entered password. The validation sets contained data for three passwords (ID = 1, 2, 3) from Table 1, and the training set with the other twelve passwords. Hence, we evaluated both SNN models on the time sequences of password entries not used in training. The data were divided into batches with the same number of positive (“same user”) and negative (“different users”) examples.

### 5.3. Evaluation Metrics

Commonly used criteria were taken to assess the quality of classifiers in the validation procedure:

- Acc—classification accuracy defined by the ratio of the number of correct users authentication to the number of all logins.
  (2) $$Acc=\frac{TP+TN}{TN+FN+TP+FP}.$$
  where TP and FP denote the number of true and false detections of hackers login, TN and FN the number of true and false predictions the accounts owners login.
- FAR—false acceptance rate
  (3) $$FAR=\frac{FP}{FP+TN}.$$
- FRR—false rejection rate
  (4) $$FRR=\frac{FN}{FN+TP}.$$
- EER—equal error rate, also known as the cross-error rate (CER), describes the point at which the false rejection rate (FRR) and false acceptance rate (FAR) are equal [28]. The lower the equal error rate value, the higher the accuracy of the biometric system. A statistic commonly used in performance evaluation of verification systems [29].
- AUC—the field under the ROC curve.

### 5.4. Snn-Based Authentication Algorithms

Finally, we developed and evaluated three variants of SNN-based algorithms, each for full and partial-password-based authentication. All algorithms aimed to classify each data sample from the input datasets into one of two categories: positive class denoted by “1” (authorized user) and negative class denoted by “0” (non-authorized user).

Firstly, we trained and validated two SNN models that were the basis of all other algorithms:

SNN(f) —a model for full-password-based authentication,
SNN(p)—a model for partial-password-based authentication.

We called them the basic classifiers. In this variant, the input of the SNN models is the current login and selected sample from collected previous logins.

In the next pair of algorithms, we assumed that the input of the SNN(f) and SNN(p) models are the current login and the average of the most recent samples (*n* previous logins). Similar to the first variant, two classifiers were developed:

SNNat(f)—a model for full-password-based authentication,
SNNat(p)—a model for partial-password-based authentication.

In the remainder of this article, we call SNN and SNNat algorithms “one-shot classification”.

In the last variant, the trained SNN models for full and partial passwords are used *n* times for input data pairs consisting of current login and the most recent previous user logins. Two classifiers were developed:

SNNn(f)—an algorithm for full-password-based authentication,
SNNn(p)—an algorithm for partial-password-based authentication.

In the remainder of this article, we call SNNn algorithms “*n*-shot classification”.

### 5.5. User Authentication Based on One-Shot Classification

**Figure 4:**
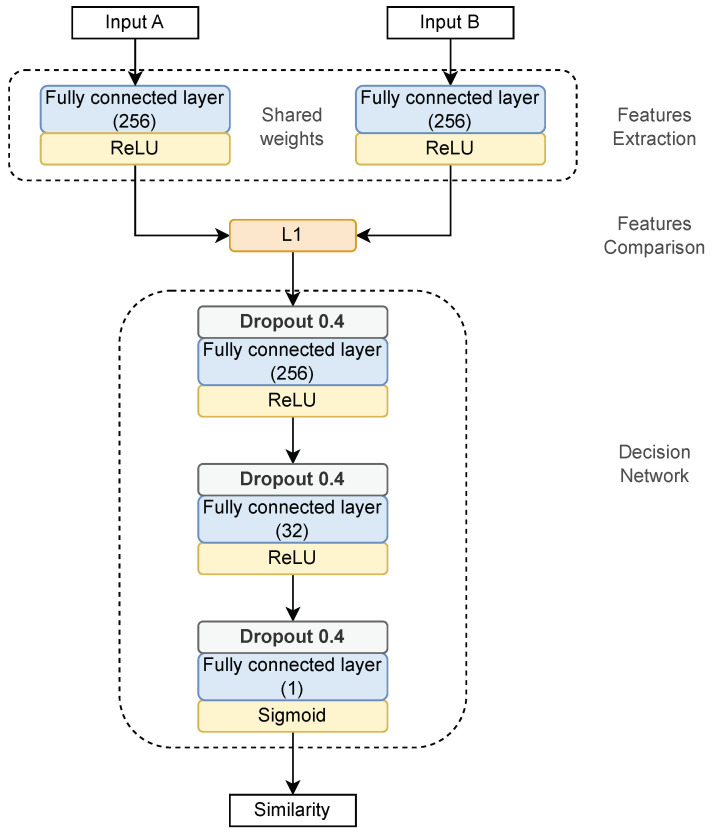
The final architecture of the Siamese neural network model.

We trained the SNN(f) classifier based on data collected from logins with full passwords. Nevertheless, training our SNN model turned out to be a challenging task. Even with a limited number of layers, our model overfitted after the first few epochs, giving unsatisfactory results. To prevent this, we added a dropout layer to the original network architecture depicted in Figure 3 (see Figure 4). Unfortunately, this was not enough. We noticed some attributes were redundant and did not significantly affect the solution. Therefore, we reduced their number to two, i.e., down-up and up-down, Figure 2. The network architecture was simplified. Finally, a full-password-driven network was trained for 300 epochs. Next, we adjusted the neural network parameters to minimize the errors (3) and (4), and maximize the classification accuracy (2) on the validation dataset.

Then we trained the SNN(p) classifier for partial passwords. We started by training the model with parameters calculated for SNN(f). Next, we tested a few architecture variants depicted in Figure 4. They differ in the number of layers, dropout value, learning rate ($l_{r}$), and weight decay ($w_{d}$). The aim was to create a model best suited to authentication based on partial passwords. The best score was obtained for $l_{r}=0.001$, $w_{d}=0.00001$. The threshold value φ for input data classification was set to 0.5.

The final accuracy of SNN(p) on validation data was equal to 66.5%. Further optimization of the network model with Optuna optimization framework, Ref. [30] resulted in an accuracy equal to 66.7%. The application of a cyclical learning rate increased the accuracy to 67.5%. Similar results were obtained for two-phase training: (i) the SNN(p) was trained on the full password dataset, and (ii) the pre-trained model was trained on the partial passwords dataset.

## 6. User Authentication Based on *n*-Shot Classification

**Figure 5:**
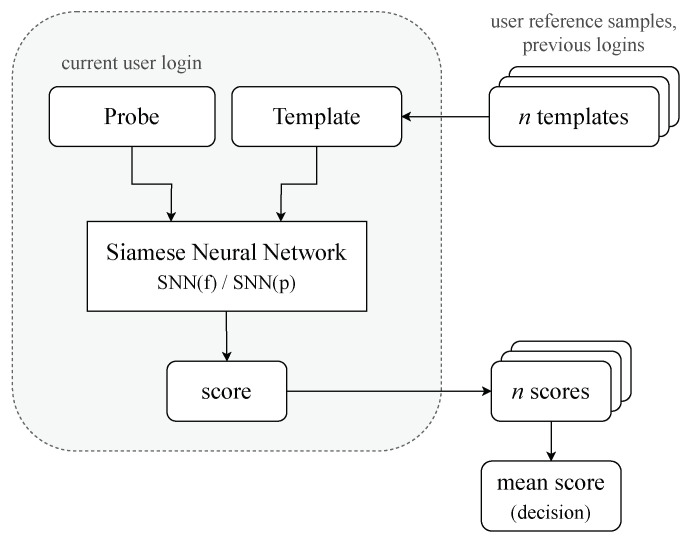
The SNNn algorithm; *n*-shot classification with *n* templates (previous logins).

The previous section presents the authentication results using pre-trained SNN(f) and SNN(p) models for the validation dataset. We call this process a one-shot classification. We conducted the next series of experiments in which we used the trained Siamese neural network model several times for input data pairs consisting of the current login and the most recent previous user logins. Thus, the authentication procedure depicted in Figure 5 consisted of the following steps:

1. For a given user, obtain reference samples from his *n* previous logins (*n* templates).
2. Create a set of SNN model inputs consisting of *n* pairs: timing distances of current login and subsequent templates.
3. Calculate the similarity score based on L1 for each pair.
4. Return the result of classification as a mean of *n* scores predictions that show the likelihood that the account holder logs on.

The experiments aimed to check how the accuracy of classifiers depends on the number of previous logins (*n*) taken into account. All experiments were conducted on validation datasets described in Section 5.2.

The results of SNN-based user authentication algorithms were compared with the tests performed on the same data using the scaled Manhattan distance for keystroke dynamics similarity calculation. The classic keystroke dynamics-based user authentication algorithms model each password as a point in *m*-dimensional space, where m=l·p is the sample size, *l* is the length of the password (number of characters), *p* the number of features considered in the timing vectors. It treats the training data as a cloud of points and computes the anomaly score of the test vector based on its proximity to the center of this cloud [14]. The timing vectors’ mean vector and each feature’s mean absolute deviation are calculated in the training phase. In the test phase, the anomaly score is calculated for each password according to the formula:

(5) $$Score=\sum_{i=1}^{m}\frac{|x_{i}-\mu_{i}|}{a_{i}},\;a_{i}=\frac{1}{n}\sum_{j=1}^{n}\left|t_{i}^{j}-\mu_{i}\right|,\;m=l\cdot p,$$

where $x_{i}$ denotes the *i*-th time distance of the probe, i.e., current login used for user authentication, $t^{j}$ denotes the *j*-th template (reference data), j=1,…,n, i.e., samples from *n* previous logins of the user, $t_{i}^{j}$ the *i*-th element of the $t^{j}$ template, $\mu_{i}$ the mean value of the *i*-th sampling element calculated based on data collected in *n* previous logins, and $a_{i}$ the average absolute deviation for *i*-th sampling element. Hence, Score is a Manhattan distance scaled by $a_{i}$.

Finally, four series of experiments were performed for full and partial passwords and the following classifiers:

SNNn(f)—*n* times used Siamese neural network (full-password-based authentication),
Scaled Manhattan(f)—the scaled Manhattan distance detector (full-password-based authentication),
SNNn(p)—*n* times used Siamese neural network (partial-password-based authentication),
Scaled Manhattan(p)—the scaled Manhattan distance detector (partial-password-based authentication).

**Figure 6:**
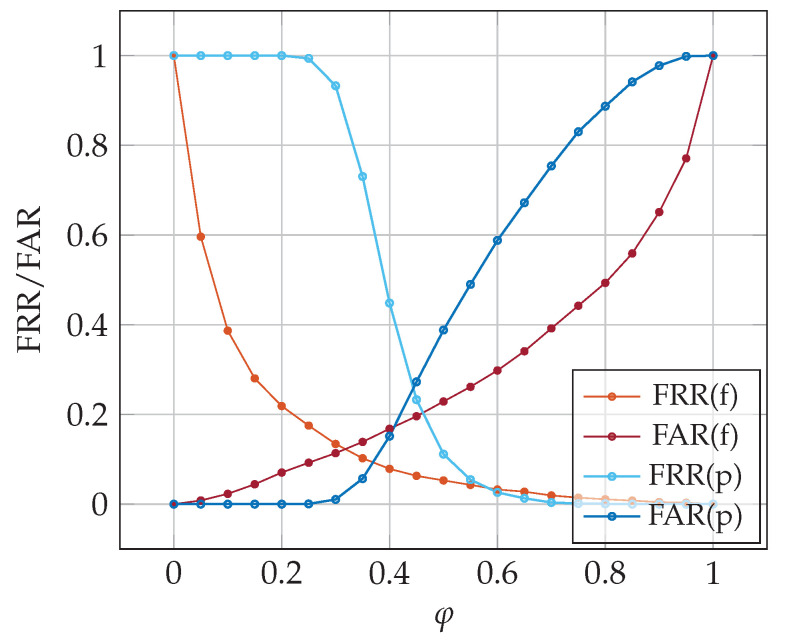
False rejection and acceptance rates for various classification thresholds; SNNn(f) and SNNn(p) algorithms, 3-shot classification.

The first series of experiments aimed to determine the threshold value φ for which the minimal equal error rate (EER) is reached. We experimented with basic SNN(f) and SNN(p) models with various φ. Figure 6 shows the false acceptance rate (FAR) and false rejection rate (FRR). Based on the experimental results presented, we selected the following threshold values

- full password authentication: $\varphi_{f}=0.30$ (FAR = 13%, FRR = 11%),
- partial password autthentication: $\varphi_{p}=0.45$ (FAR = 23%, FRR = 27%).

**Figure 7:**
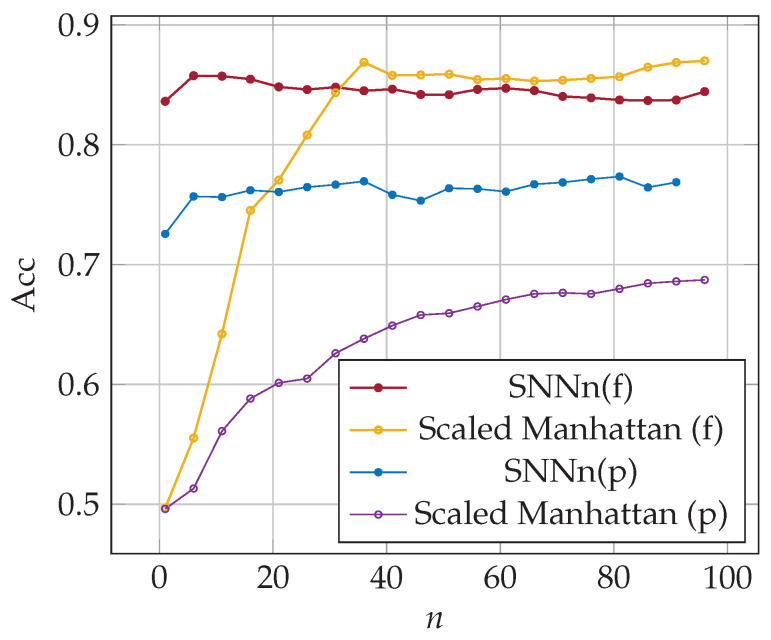
Accuracy of SNNn algorithms and the baseline method; reference data from all previous logins ($\varphi_{f}=0.30$, $\varphi_{p}=0.45$).

Experiments were then carried out to investigate how data extracted from previous logins affected the accuracy of the classifiers. The results are depicted in Figure 7. It can be seen that for both classifiers employing Siamese networks, the accuracy seriously increases only for the first few logins considered. The inclusion of more historical data slightly affects the accuracy of the classification. The best accuracy for the SNNn(f) was equal to 86% using reference data from 5 previous logins, while the best accuracy for the SNNn(p) was equal to 77% using 38 past logins.

As was already pointed the usage of partial password challenges keystroke dynamics-based authentication; hence the classification accuracy is lower for this approach, as presented in Figure 7. In the case of partial passwords, each time, different characters are selected for the user to enter, so the time sequences of subsequent logins are not the same. We assumed that the user types characters at a similar pace, regardless of the distance between the characters in the password. Although our SNNn(p) classifier does not use information about the position of the typed character, it gives the best results from the approaches evaluated in this research.

In the case of the baseline method, results significantly depend on the number of data samples from past logins, both in the case of full and partial password authentication. The baseline method achieved the same accuracy as the SNNn(f) classifier for the full password after 32 logins. Its accuracy increased further, reaching a maximum of 87% with 36 samples. The conclusion is that for a large enough dataset containing login data, the classification quality using the simple basic method and a Siamese network could be similar in the case of a full password authentication procedure. The results are different for partial password authentication. The classification accuracy for the baseline method was equal to 69% after 96 logins, while SNNn(p) gave 77% for 38 past logins.

Some authentication systems recommend that users change their password every assumed time (e.g., every month). It can significantly affect the quality of the baseline method, both in the case of full and partial passwords. It can produce inaccurate results due to the inefficient number of collected data about logins. The SNNn algorithm should give acceptable results even for a small number of past logins *n* with the new password.

## 7. Experimental Performance Evaluation of Authentication Methods

### 7.1. Experiment Setup and Datasets

Finally, SNNn(f) and SNNn(p) classifiers were used for online user authentication. Security best practice suggests changing the password every month, so we focused on recognizing the user by a small number of previous logins. A series of experiments were conducted for passwords and data about keystroke dynamics that were not used in SNN architecture training and validation. Participants in the experiment created new passwords that they could not learn beforehand. They are collected in Table 3.

We changed data acquisition rules to make our tests as realistic as possible. First of all, login credentials were not distributed among participants. Each user had to sign in and create an account. Therefore, when logging in to another (foreign) account, the users used a password they did not know before, i.e., they entered it for the first time. Second, participants were asked to log in once daily to avoid mechanical typing and unrealistically similar keystroke dynamics during login. Thirteen volunteers, working with computers daily, aged between 26 and 40 years, participated in the experiment that lasted 13 days.

The scenario of the experiment was as follows. Participants logged in using our data acquisition tool described in Section 5.1. They chose their username and password themselves at the registration stage. The only restriction was the number of characters of the password, which could not be less than 8. After 13 days, the phase of collecting valid logins ended, and the main part of the experiment, i.e., logging into other study participants’ accounts, began. In the following 12 days, each user received an email with the login and password of the person to whose account they were to log in (as a hacker). Our classification algorithms, i.e., SNNn(f) or SNNn(p), were used to identify the user for every login. The threshold values were set to $\varphi_{f}=0.30$ and $\varphi_{p}=0.45$. The number of reference samples from previous logins (*n*) was 1 to 12.

### 7.2. Comparison Baselines

The results of SNNn classifiers were compared with the tests performed on the same data using simple base methods. We used the scaled Manhattan distance described in Section 6, the Mahalanobis distance, and the Instance-based Tail Area Density (ITAD), as the baselines.

#### 7.2.1. Mahalanobis Distance

The algorithm described in [31] uses the Mahalanobis distance for login samples comparison. The Mahalanobis distance measures the distance between a point *x* and a distribution *Q*. It can be seen as an extension of the Euclidean distance to account for correlations between features [14]. The score is calculated as the distance between the mean vector, calculated from the collected data, and a given test vector:

(6) $$Score=\frac{1}{m}\sqrt{\sum_{i=1}^{m}\frac{{\left(x_{i}-\mu_{i}\right)}^{2}}{\sigma_{i}^{2}}},$$

where *m* is the number of time distances shared between the probe sample and the template, $x_{i}$ is the *i*-th time distance of the test sample, and $\mu_{i}$ and $\sigma_{i}$ are the mean and standard deviation of the *i*-th feature, calculated from the collected data.

#### 7.2.2. ITAD Distance

The Instance-based Tail Area Density (ITAD) metric, proposed in [17], is based solely on the tail area under the sample’s PDF (Probability Density Function) or percentile value. Assuming that the probe sample and templates are time vectors recorded while entering the same password, the ITAD metric is calculated as follows:

(7) $$s_{i}=\left\{\begin{array}{cc} CDF_{i}\left(x_{i}\right), & x_{i}<=M_{i},\\ 1-CDF_{i}\left(x_{i}\right), & x_{i}>M_{i}, \end{array}\right.$$

where $CDF_{i}\left(\cdot \right)$ is the empirical cumulative distribution function of the *i*-th time distance of templates, $M_{i}$ is the median of the *i*-th time distance of templates, $x_{i}$ is the *i*-th time distance of the probe, and *m* is the number of features (time distances) in the test sample and templates. The similarity score for *m* time distances is an average of ITAD scores calculated for all features:

(8) $$Score=\frac{1}{m}\sum_{i=1}^{m}s_{i}.$$

### 7.3. Results of Experiments

**Figure 8:**
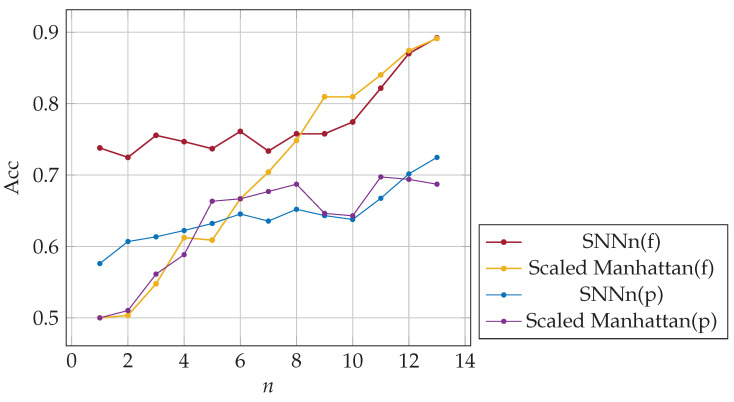
The accuracy of SNNn and baseline algorithms; *n*-shot classification on full (f) and partial (p) passwords.

**Figure 9:**
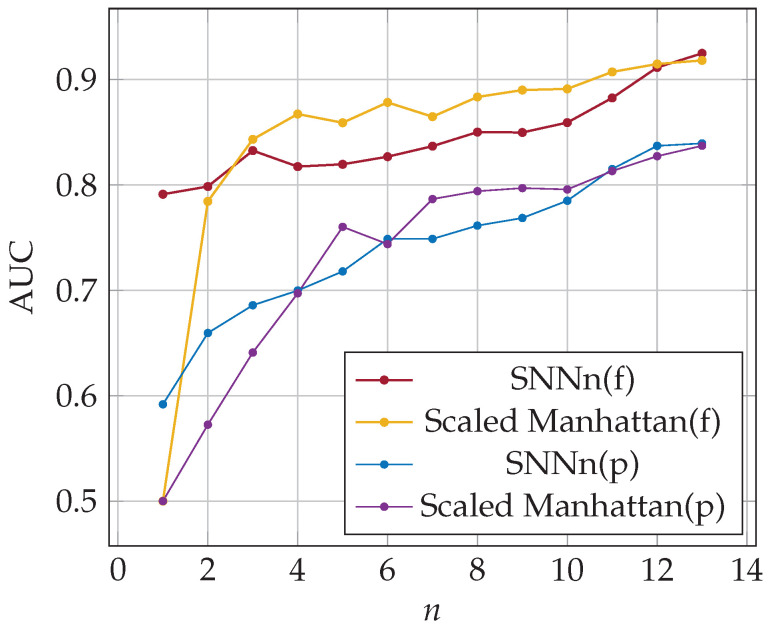
The area under the ROC curve for the SNNn algorithms; various numbers of templates (previous logins).

The metrics described in Section 5.3 were used for the performance evaluation of tested classifiers. False acceptance and rejection rates after three logins for full and partial-password-based authentication were as follows: FAR(f) = 0.25, FAR(p) = 0.32, FRR(f) = 0.28, FRR(p) = 0.36. The minimal equal error rate for SNNn(p) classifier was similar to once obtained for the validation dataset, ERR(p) = 0.3. In the case of full-password-based authentication, the value of ERR was worse w.r.t. the obtained for validation dataset, i.e., ERR(f) = 0.27. Hence the classification quality was slightly worse. The classification accuracy as a function of the number of past logins *n* used in the authentication procedure is presented in Figure 8, the area under the ROC curve (AUC) in Figure 9. Note that the user learns their password in the first few logins, and how they write it may change dynamically until the habit is established. Verifying a user after several logins is difficult due to user typing changes. Nevertheless, SNN(f) classification accuracy was about 74% after one login and 89% after twelve logins. The results of the SNNn(p) classifier were significantly worse but still outperformed the results of the baseline method, especially for a few previous logins. After twelve past logins, the classification accuracy was about 73%. Generally, the quality of classification both for SNNn(f) and SNNn(p) increases with the number of reference samples (logins) considered, and the potential for further growth can be seen.

It can be seen comparing the results shown in Figure 7 and Figure 8 that when passwords are changed, and users are just learning them, there is an opportunity after a few logins for more effective authentication, mainly based on full passwords. Users are likely more straightforward for the classifier to distinguish. Hence, we recommend inducing users to change their passwords, especially for automatic authentication based on SNNs and full passwords.

Table 4 and Table 5 show the authentication results of all experiment participants. It can be seen that the accuracy of authentication is significantly different for different users. In some cases, SNNn(f) and SNNn(p) could not classify the input data correctly, or the classification accuracy was very low. Unfortunately, a thorough analysis of the input data did not indicate what might be causing the problems. It is likely that the amount of data collected to learn the model needed to be larger or that the passwords’ dynamics during successive logins varied too much for the network to detect characteristic features. For other users, the classification results were outstanding.

**Figure 10:**
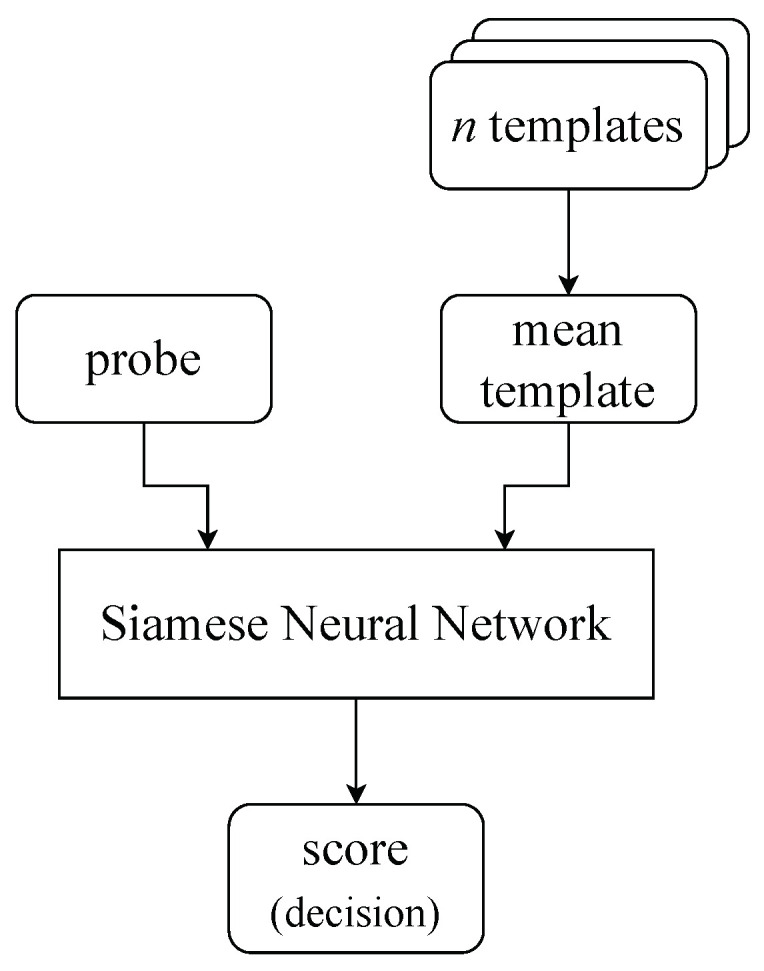
The SNNat algorithm—usage of the average template based on *n* previous logins.

**Figure 11:**
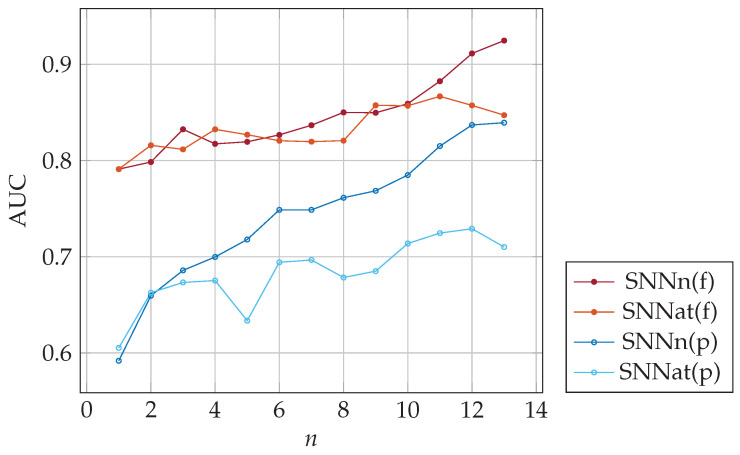
The area under the ROC curve for the SNNn and SNNat algorithms; various numbers of templates (previous logins).

The classifiers SNNn(f) and SNNn(p) require *n* SNN model responses, which take some time. In the last series of tests, we evaluated another approach (SNNat), presented in Figure 10. During each user login, the dynamics of the password entry were compared with a template calculated as the average of the input data from *n* previous logins. Thus, the one-shot classification with an average template was employed. The comparative study of SNNn and SNNat classifiers is presented in Figure 11. It can be seen that SNNn-type classifiers give much better results. The area under the ROC curve (AUC) significantly decreases for SNNat and full-password-based authentication. The reduction in classification quality in authentication on partial passwords is significantly less, but the SNNn algorithm still gives better results than SNNat. In conclusion, we recommend the use of SNNn-type algorithms.

Finally, it is worth noting that comparing the effectiveness of user authentication methods proposed by different authors is usually impossible. The results depend on the training and validation datasets, size, and the data acquisition procedure. Unfortunately, algorithms are trained on different datasets that are usually not publicly available. Therefore, we could not present the comparative study of full-password-based authentication algorithms described in [4,16] with our Siamese network-based techniques for full passwords. All investigated classifiers, i.e., our algorithms and reported in the literature, were trained, validated, and tested on different datasets. Moreover, the data acquisition procedure was different. For example, the CMU dataset [14] used in [16] collects the data of individuals who entered the same password on the same machine. Not all the mentioned datasets and trained network models are available online. Therefore, a comparative study of methods under the same conditions was impossible.

### 7.4. Improved SNN Models

Since we expected better results from the neural network, we decided to retrace the learning process to make potential improvements. We made some rather challenging assumptions in training the networks SNN(f) and SNN(p). First, we assumed the network would learn to compare the dynamics of passwords entered by users other than those sampled in the training process. Second, in acquiring the data forming the training set, false logins were entered as many times as correct logins. Thus, the network could not learn to recognize the attacker by the time interval the password was entered. It is expected that he will type the password more slowly. Therefore, we modified the training set and created new pairs of “same” and “different” samples than we did previously (see Algorythm 1).

**Figure 12:**
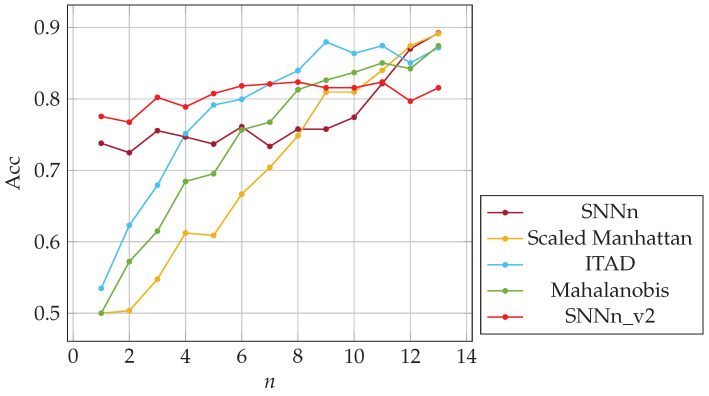
The accuracy of SNN-based and baseline algorithms; *n*-shot classification on full passwords.

**Figure 13:**
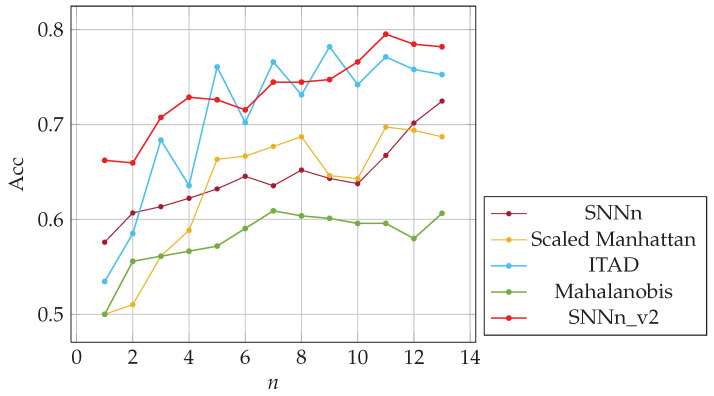
The accuracy of SNN-based and baseline algorithms; *n*-shot classification on partial passwords.

For each sample of a given user, we created a positive pair with subsequent sample of the same user and a negative pair with a random sample of another user entering the same password. It ensured that the “same” samples were created from keystroke dynamics more similarly. We trained and validated both SNNs on the new training dataset. We obtained new models, i.e., SNNn_v2(f) and SNNn_v2(p). Finally, we implemented these new network models in our *n*-shot classification algorithms. We evaluated both algorithms, i.e., SNNn_v2(f) and SNNn_v2(p), on the test dataset described in Section 7.1, and compared with original SNNn(f) and SNNn(p), and baseline methods. The results are presented in Figure 12 and Figure 13. New Siamese network models allowed us to achieve better accuracy in comparison to the original ones used in the experiments presented so far.

It is worth noting how data preprocessing affected the accuracy of the model. The network trained with the second approach of creating sample pairs (SNNn_v2) is more effective than the previous variant. Compared to the baseline methods, SNNn_v2 yields much better results for a small number of user templates (previous logins). For a larger number of templates, it performs comparably (partial passwords) or worse (full passwords) w.r.t. the baselines methods.

In conclusion, based on the results of experiments, we recommend a hybrid system for keystroke dynamics-based authentication that combines two techniques:

- SNNn algorithms until the required number of login samples is obtained;
- A selected simple algorithm based on distances between samples calculation after collecting an adequate number of login samples.

The switching between algorithms has to be experimentally determined.

## 8. Conclusions

This paper presents algorithms employing Siamese neural network models and *n*-shot classification for static keystroke dynamics-based user authentication on full and partial passwords. Our network architecture was trained, validated, and tested on real keystroke datasets containing samples for full and partial passwords. Several variants of our algorithms differ in network architecture, and parameters were simulated and compared. The final results of our research are two modules, i.e., the data acquisition module and SNN-based algorithms for user authentication on full and partial passwords. We have focused our attention on the use of partial passwords. According to our knowledge, there are no other methods to deal with the problem of partial password behavioral biometrics.

We compared the performance of our authentication algorithms with classical techniques using three metrics: scaled Manhattan, Mahalanobis, and Instance-based Tail Area Density (ITAD). In general, our solution gave satisfactory results outperforming the baseline, especially when only a few enrollment samples were available. It should be noted that the simple classification method based on distances between samples calculation cannot be used immediately after changing the password. We need at least two measurements (two logins) to return a meaningful result. Moreover, numerical experiments show that the accuracy of this method seriously depends on the number of trials and is only effective after dozens of previous logins. More data require increased computational complexity, affecting the time to calculate the anomaly score.

In conclusion, we recommend using algorithms based on Siamese neural network models and *n*-shot classification as a tool to support user authentication and increase users’ confidence. After a sufficiently large number of logins, we recommend switching to a simple algorithm based on the chosen metric of counting the distance between samples. It should be noted, however, that when designing an authentication mechanism based on keystroke dynamics, it is crucial to be aware that all solutions that attempt to model a specific user have less durability and need to be retrained, as typing dynamics can change over time.

In our future work, we plan to train and evaluate our network models on more diverse datasets of samples from people of different age groups and computer skills. Gathering data for neural network model training is a challenge. In our research, we gathered volunteers among people who work at a computer daily. With a more diverse set of users, the differences in their typing speed will be more significant, and the accuracy of distinguishing between them should also be more significant. The second research area under consideration is to build neural network models that model the behavior of individual users and test if they significantly improve classification quality.

## Figures and Tables

**Table 1:** The passwords used in the experiments.

Password Id	Password	Password Length
1	V4f@eyerik	10
2	1A_!MOr1si	10
3	W#aZEwA@7d	10
4	cru@wo9Ac9	10
5	Bat3eb-Chu	10
6	2OPR@wrunI	10
7	5l&w&CHaza	10
8	SO@o&RLqL2	10
9	fobR+spi2l	10
10	!TU#usUs1O	10
11	crosp0Z*	8
12	KoszulkaPolo:12	15
13	5,HaveFun	9
14	Van_Helsing15	13
15	ZlotyCzlowiek1.	15

**Table 2:** The example pairs of the SNN model input vectors.

Character Number	Key Event	Sample 1 [s]	Sample 2 [s]
1	down-up	0.0117	0.0063
	up-down	0.1414	0.2526
2	down-up	0.0090	0.0067
	up-down	0.0563	0.2056
3	down-up	0.0137	0.0139
	up-down	0.1370	0.1310
4	down-up	0.0173	0.0115
	up-down	0.1552	0.1185
...	...	...	...

**Table 3:** Testing dataset–passwords created by users.

Password Id	Password	Length
1	vCpZq9cgS24jxp	14
2	D1ub13S0b13%&*	15
3	TegPe$ak16	10
4	PjPaWdSt@6x3	12
5	Ko1Ne@WKa#	10
6	DCS2Sc@da14	11
7	kurkabela	9
8	ArtzBz8zc	9
9	wGh58L12	8
10	neutrino	8
11	a!S2D3f$	8
12	CzyTo1358??	11
13	goscu1999	9

**Table 4:** The experiment participants’ authentication efficiency after three logins (full password).

User	Precision	Recall	FRR	FAR	Acc
1	0.75	0.23	0.07	0.77	0.58
2	0.92	1.0	0.08	0.0	0.96
3	0.97	0.83	0.03	0.16	0.90
4	0.63	0.33	0.2	0.67	0.56
5	0.80	0.62	0.15	0.38	0.73
6	1.0	0.42	0.0	0.58	0.70
7	1.0	0.92	0.0	0.08	0.96
8	0.61	1.0	0.63	0.0	0.68
9	0.5	1.0	1.0	0.0	0.5
10	0.71	0.83	0.33	0.16	0.75
11	0.6	0.75	0.5	0.25	0.63
12	0.8	0.57	0.14	0.43	0.71
13	0.59	1.0	0.69	0.0	0.65

**Table 5:** The experiment participants’ authentication efficiency after three logins (partial password).

User	Precision	Recall	FRR	FAR	Acc
1	0.88	0.53	0.08	0.46	0.73
2	0.54	0.5	0.42	0.5	0.54
3	0.56	0.14	0.11	0.86	0.51
4	0.82	0.60	0.13	0.40	0.73
5	0.75	0.23	0.08	0.77	0.58
6	1.0	0.58	0.0	0.42	0.79
7	1.0	0.83	0.0	0.17	0.92
8	0.78	0.58	0.16	0.42	0.71
9	0.90	0.75	0.08	0.25	0.83
10	0.50	0.17	0.17	0.83	0.50
11	0.40	0.33	0.5	0.67	0.42
12	0.83	0.36	0.07	0.64	0.64
13	0.78	0.54	0.15	0.46	0.69

## Data Availability

Data supporting reported results are available on request from the corresponding author.
